# Divergent Evolution of Mutation Rates and Biases in the Long-Term Evolution Experiment with *Escherichia coli*

**DOI:** 10.1093/gbe/evaa178

**Published:** 2020-08-27

**Authors:** Rohan Maddamsetti, Nkrumah A Grant

**Affiliations:** e1 Department of Biomedical Engineering, Duke University; e2 BEACON Center for the Study of Evolution in Action, Michigan State University; e3 Department of Microbiology and Molecular Genetics, Michigan State University; e4 Program in Ecology, Evolutionary Biology and Behavior, Michigan State University

**Keywords:** experimental evolution, metagenomics, mutation

## Abstract

All organisms encode enzymes that replicate, maintain, pack, recombine, and repair their genetic material. For this reason, mutation rates and biases also evolve by mutation, variation, and natural selection. By examining metagenomic time series of the Lenski long-term evolution experiment (LTEE) with *Escherichia coli* (Good BH, McDonald MJ, Barrick JE, Lenski RE, Desai MM. 2017. The dynamics of molecular evolution over 60,000 generations. Nature 551(7678):45–50.), we find that local mutation rate variation has evolved during the LTEE. Each LTEE population has evolved idiosyncratic differences in their rates of point mutations, indels, and mobile element insertions, due to the fixation of various hypermutator and antimutator alleles. One LTEE population, called Ara+3, shows a strong, symmetric wave pattern in its density of point mutations, radiating from the origin of replication. This pattern is largely missing from the other LTEE populations, most of which evolved missense, indel, or structural mutations in *topA*, *fis*, and *dusB*—loci that all affect DNA topology. The distribution of mutations in those genes over time suggests epistasis and historical contingency in the evolution of DNA topology, which may have in turn affected local mutation rates. Overall, the replicate populations of the LTEE have largely diverged in their mutation rates and biases, even though they have adapted to identical abiotic conditions.

**Significance**

Bacteria often evolve elevated mutation rates during adaptation to challenging environments. Less is known about how mutation rates vary over the chromosome, and how those local biases evolve during adaptive evolution. To answer this question, we analyzed metagenomic data from an ongoing experiment with *Escherichia coli* in which 12 replicate populations of bacteria, started from a single clonal strain in 1988, were allowed to evolve for more than 30 years. We find that each replicate population has a different genomic distribution of observed mutations, indicating that local mutation rates have evolved idiosyncratically, even though each population has adapted to the same laboratory conditions. Intriguingly, our results indicate that adaptive mutations that change DNA topology may also affect local mutation rates.

## Introduction

Loci that modify DNA repair and recombination modify the evolutionary process. Therefore, one might ask whether natural selection adaptively tunes mutation and recombination rates. This idea—that second-order selection *adaptively* modifies the evolutionary process itself—is debated (Tenaillon et al. 2001; Lynch et al. 2016). Nonetheless, populations of *Escherichia coli*, engineered to have constitutive sexual recombination and elevated mutation rates, adapt faster than control populations in the laboratory (Cooper 2007; Peabody et al. 2016, 2017).

To study second-order selection on mutation rates, one can use experimental evolution. By running experiments in which replicate populations evolve under controlled conditions, with different starting mutation rates, one can ask whether particular mutation rates are favored over others (Chao et al. 1983; Loh et al. 2010; Sprouffske et al. 2018). Here, we use metagenomic time series data from the Lenski long-term evolution experiment (LTEE) with *E. coli* to study how mutation rates evolve in real time.

In the LTEE, 12 populations of *E. coli*, descended from a common ancestral strain, have adapted for more than 73,000 generations to carbon-limited minimal media. Six of the populations are labeled Ara+, whereas the other six are labeled Ara−, based on the presence or absence of an evolutionarily neutral arabinose marker (Lenski et al. 1991). The LTEE populations are strictly asexual. Some populations have evolved defects in DNA repair which vastly increase their point mutation rates. The causative hypermutator alleles likely went to fixation by linkage with highly beneficial mutations, rather than being beneficial per se (Sniegowski et al. 1997; Tenaillon et al. 2016). We refer to the LTEE populations that have evolved large increases in point mutation rates as “hypermutator populations,” and refer to the others as “nonmutator populations.”

Molecular evolution in the hypermutator populations of the LTEE is dominated by “genetic draft,” in which large numbers of nearly neutral passenger mutations hitchhike with a small number of beneficial driver mutations (Neher 2013). This phenomenon has obscured the genomic signatures of adaptation in those populations (Tenaillon et al. 2016; Couce et al. 2017; Good et al. 2017; Maddamsetti et al. 2017). In this regime, also called “emergent neutrality” (Schiffels et al. 2011), the evolutionary dynamics inferred from whole-population samples of the hypermutator populations (Good et al. 2017) provides good data on mutation rates and biases, even though natural selection drives the dynamics. Here, we examined LTEE metagenomics data (Good et al. 2017) for mutation rate variation and biases over the chromosome (Foster et al. 2013; Paul et al. 2013; Jee et al. 2016; Niccum et al. 2019).

## Results

### Cumulative Number of Observed Mutations in Each Population Reveals Dynamics Caused by Both Hypermutator and Antimutator Alleles

**Fig. 1.**
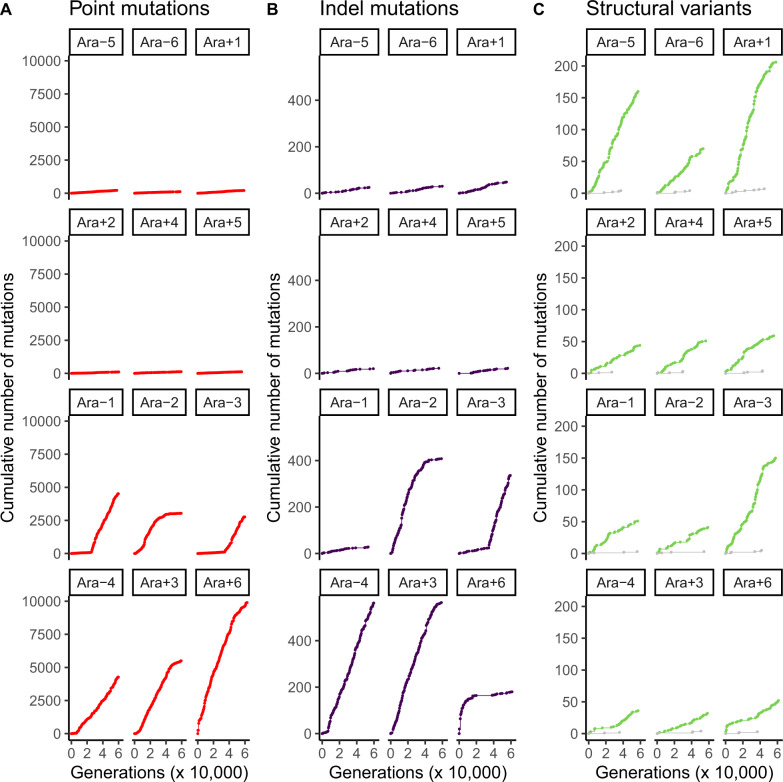
Divergent evolution of mutation rates in the LTEE. Each panel shows the cumulative number of observed mutations, subdivided by mutation class, over time in each LTEE population. The top six panels show the nonmutator LTEE populations, and the bottom six panels show the hypermutator LTEE populations. (*A*) Point mutations are shown in red. (*B*) Indel mutations are shown in purple. (*C*) sv associated with transposons are shown in green, whereas those that are not associated with transposons are shown in gray.

**Fig. 2.**
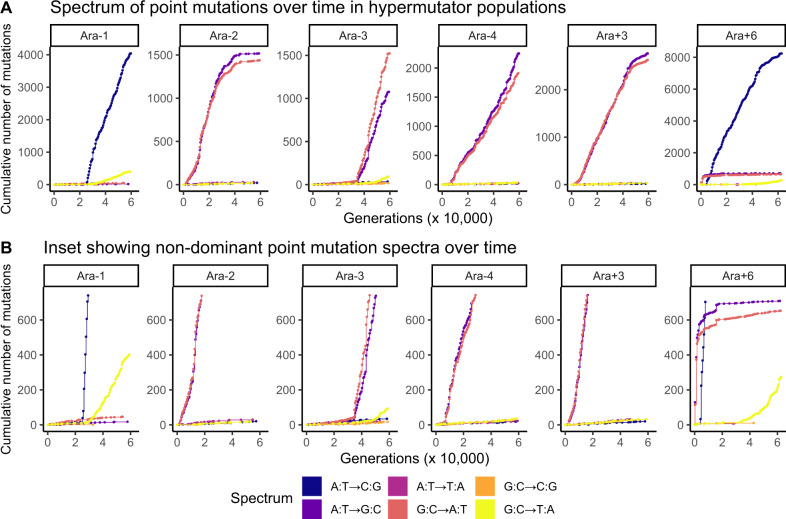
The dynamics of hypermutator and antimutator alleles affect the spectrum of observed point mutations over time. (*A*) Spectrum of point mutations over time in the hypermutator LTEE populations. (*B*) Inset figure showing nondominant point mutation spectra over time in the hypermutator LTEE populations.

We examined the number of observed mutations over time in each LTEE population (figs. 1 and 2, supplementary figs. S1–S3, Supplementary Material online). These results show that mutation rates have evolved idiosyncratically over the LTEE. Figure 1*A* shows the number of point mutations over time in each population. The rate of observed point mutations decreased in three of the six hypermutator populations (Ara−2, Ara+3, and Ara+6). The decrease in the rate of molecular evolution in these populations was previously ascribed to the evolution of antimutator alleles (Tenaillon et al. 2016; Good et al. 2017). Although antimutator alleles of *mutY* compensating for defects in *mutT* have been reported in Ara−1 (Wielgoss et al. 2013), the change in slope observed at 40,000 generations in Ara−1 is subtle compared with the slope changes in Ara−2, Ara+3, and Ara+6.

**Fig. 3.**
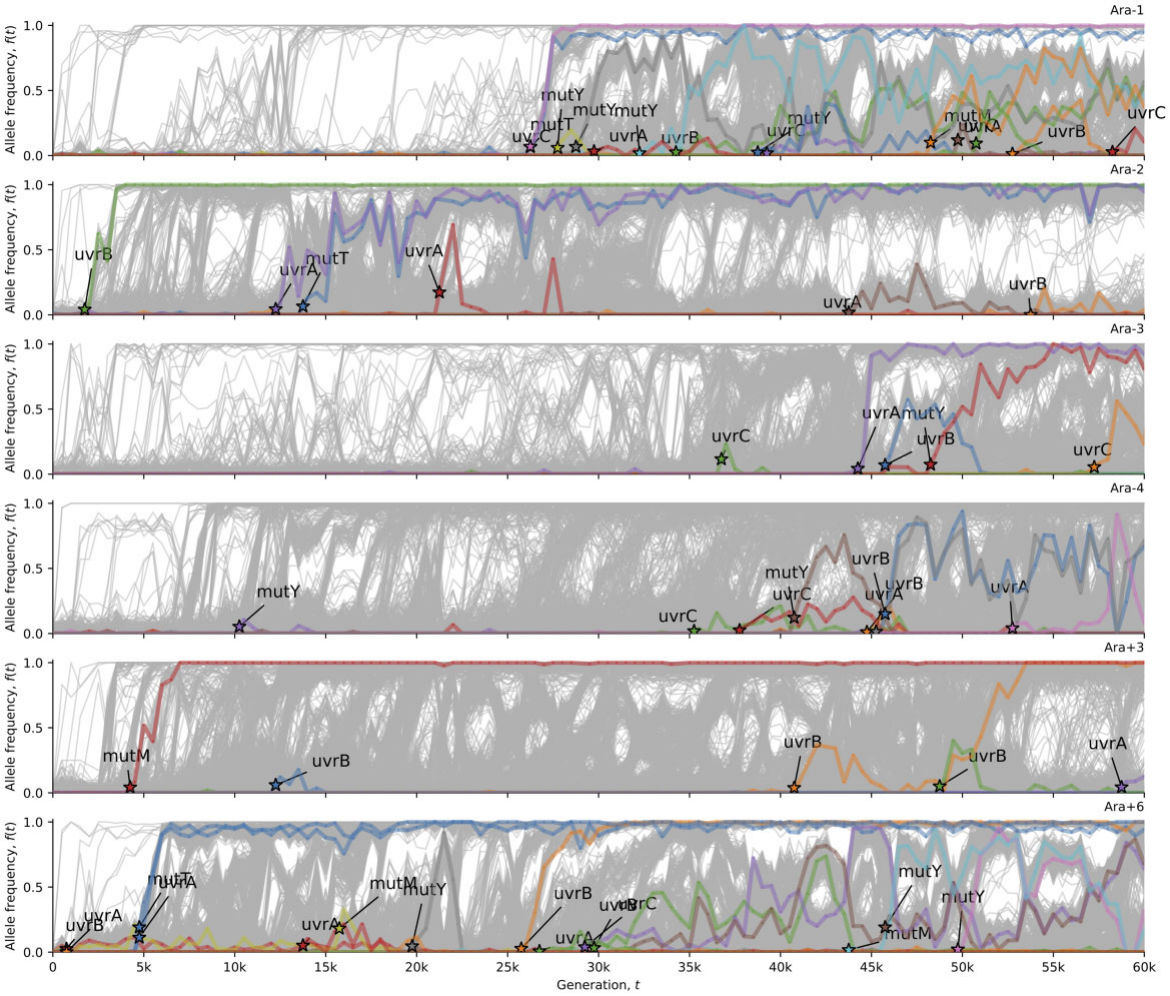
Oxidative damage repair alleles in hypermutator LTEE populations. This visualization uses computer code from Good et al. (2017). Stars indicate the time (and allele frequency) at which mutations are reliably estimated to appear in the time series. The allele frequency trajectories for all observed mutations in the hypermutator populations are shown in gray. The allele frequency trajectories of de novo mutations (excepting synonymous mutations) in oxidative damage repair genes (supplementary file 1, Supplementary Material online) are colored and labeled in each population.

**Fig. 4.**
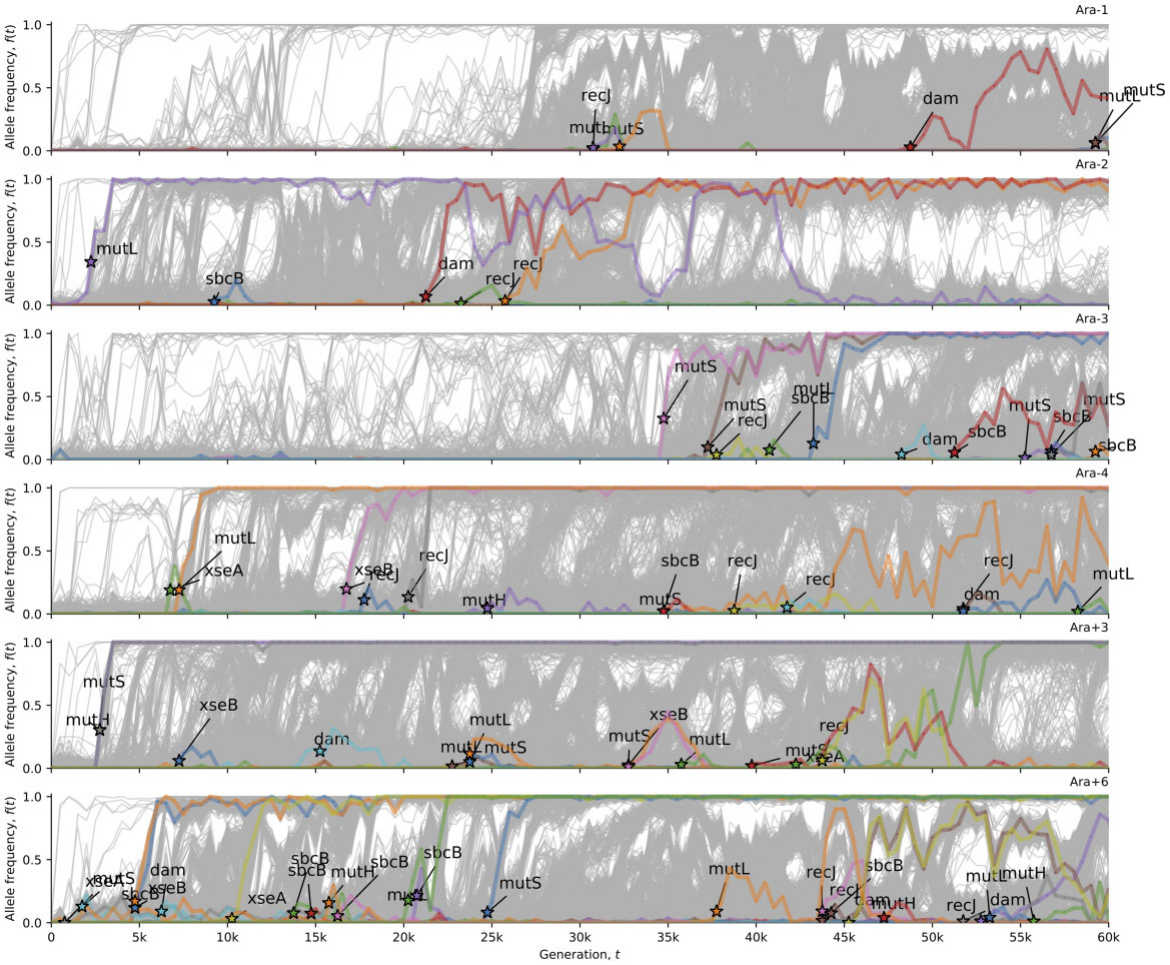
MMR alleles in the hypermutator LTEE populations. This visualization uses computer code from Good et al. (2017). Stars indicate the time (and allele frequency) at which mutations are reliably estimated to appear in the time series. The allele frequency trajectories for all observed mutations in the hypermutator populations are shown in gray. The allele frequency trajectories of de novo mutations (except synonymous mutations) in MMR genes (supplementary file 1, Supplementary Material online) are colored and labeled in each population.

Figure 1*B* shows the number of observed indel mutations over time in each population. Five of the six point-mutation hypermutator populations also show an indel hypermutator phenotype. These five populations all evolved defects in mismatch repair (MMR) (table 1 and fig. 4). The exception is Ara−1, which evolved a frameshift *mutT* allele (table 1 and fig. 3) that induces a high point mutation rate, absent a corresponding indel hypermutator phenotype.

**Table 1.** Putative Hypermutator and Antimutator Alleles Described in the Text

Population	Gene	DNA Repair Pathway	Appearance Time (Generations)	Position (bp)	Mutation
Ara−1	*uvrC*	Oxidative damage repair	26,250	1,972,086	Q183P
Ara−1	*mutT*	Oxidative damage repair	26,250	114,034	(C)_6→7_
Ara−1	*mutY*	Oxidative damage repair	28,750	2,988,792	L40W
Ara−1	*mutY*	Oxidative damage repair	32,250	2,989,164	L164*
Ara−2	*mutL*	MMR	2,250	4,375,786	(TGGCGC)_3→4_
Ara−2	*uvrA*	Oxidative damage repair	12,250	4,251,585	A407T
Ara−2	*mutT*	Oxidative damage repair	13,750	114,113	R89H
Ara−2	*mutL*	MMR	*This in-frame reversion fixes at 42,250 generations	4,375,781	(TGGCGC)_3→2_
Ara−3	*mutS*	MMR	34,750	2,753,768	Q606*
Ara−3	*mutY*	Oxidative damage repair	48,250	2,989,624	Δ1 bp
Ara−4	*mutL*	MMR	7,250	4,375,781	(TGGCGC)_3→2_
Ara+3	*mutS*	MMR	2,750	2,752,473	+G
Ara+6	*mutS*	MMR	1,250	2,752,473	+G
Ara+6	*uvrA*	Oxidative damage repair	4,750	4,250,341	I821M
Ara+6	*mutT*	Oxidative damage repair	4,750	114,034	(C)_6→5_
Ara+6	*mutY*	Oxidative damage repair	31,750	2,988,917	Y82D
Ara+6	*mutY*	Oxidative damage repair	49,750	2,989,297	C208W

The hypermutator dynamics in Ara−2 are particularly striking. An antimutator allele eventually fixes, and reverts both the point and indel hypermutator phenotype back to ancestral or near ancestral levels (fig. 1*A* and *B*). The hypermutator phenotype is caused by phase variation of a (TGGCGC)_3_ repeat in *mutL* (table 1). Reversions to the triplet state reverse the hypermutator phenotype. The number of new point and indel mutations in Ara−2 (supplementary figs. S1 and S2, Supplementary Material online) fluctuates with the allele frequency dynamics of this *mutL* repeat (fig. 4). Although fixations are usually irreversible in large asexual populations, phase variation is an exception: polymerases often slip on repetitive sequences, causing those repeats to expand or contract at relatively high rates (Moxon et al. 2006).

At first glance, figure 1*B* seems to show that Ara+6 fixed a mutation reverting the indel hypermutator phenotype. However, a close examination of the indel mutation rate and allele frequency dynamics in Ara+6 reveals that a super-hypermutator clade evolved within the first 1,000 generations (supplementary fig. S2, Supplementary Material online). Additional evidence for the super-hypermutator clade comes from the evolution and extinction of an A:T→G:C and G:C→A:T hypermutator phenotype (fig. 2) that parallels the evolution of the indel hypermutator phenotype. This super-hypermutator clade carries a frameshift allele of the MMR gene *mutS* (table 1 and fig. 4), is distinguished by marker alleles of the nucleotide excision repair genes *uvrA* and *uvrB* (fig. 3), and persists at low frequency until going extinct by 20,000 generations (figs. 3 and 4, supplementary fig. S2, Supplementary Material online). The majority clade in Ara+6 evolved a mutation in *mutT* at 4,750 generations (table 1 and fig. 3) that causes a point mutation hypermutator phenotype without causing an indel hypermutator phenotype. The coexistence of clades with different hypermutator phenotypes, and the eventual extinction of the super-hypermutator clade, most reasonably explains the loss of the indel hypermutator phenotype from Ara+6.

Figure 1*C* shows the number of observed structural mutations over time. As described in the original report for this data set (Good et al. 2017), structural mutations (or structural variants, sv) are defined by junctions between two distinct locations in the reference genome. The vast majority of these structural mutations are caused by insertion sequence (IS) transpositions. Three of the canonical nonmutator populations (Ara−5, Ara−6, and Ara+1) show an IS hypermutator phenotype. The IS hypermutator phenotype in Ara+1 was reported previously (Papadopoulos et al. 1999; Tenaillon et al. 2016). In contrast, only one of the canonical hypermutator populations, Ara−3, shows an IS hypermutator phenotype. The rate of observed structural mutations in Ara−3 shows three different slopes. Ara−3 evolved an IS hypermutator phenotype very early in the LTEE. Around 30,000 generations, the IS rate intensifies, either due to genetic evolution, or as a consequence of stress induced by the citrate metabolic innovation that evolved around that time (Blount et al. 2012, 2020). Finally, the IS rate decreases around 45,000 generations. More than 100 mutations go to fixation in the selective sweep at 45,000 generations in Ara−3, including mutations in the DNA repair genes *recR*, *recE*, *ligA*, *uvrA*, and *ybaZ*. The distinct IS rates observed in Ara−3 may, in part, reflect clonal interference between deeply diverged, competing lineages in that population (Blount et al. 2012; Leon et al. 2018), especially if those lineages have different IS transposition rates.

We also examined the spectrum of point mutations in each hypermutator population over time (fig. 2). Ara–1 and Ara+6 show a high frequency of A:T→C:G transversion mutations, characteristic of defects in *mutT* (Tajiri et al. 1995; Fowler et al. 2003; Wielgoss et al. 2013). Ara–2, Ara–3, Ara–4, and Ara+3, which all have defects in MMR (table 1 and fig. 4), show a high frequency of A:T→G:C and G:C→A:T mutations. These findings are consistent with genomic analyses of LTEE hypermutators (Couce et al. 2017). Furthermore, Ara−1, Ara−3, and Ara+6 all show late increases in the frequency of G:C→T:A transversion mutations, characteristic of defects in *mutY* (Tajiri et al. 1995; Fowler et al. 2003; Wielgoss et al. 2013).

In examining *mutT*, we noticed that two of the three cases of *mutT* alleles arising to high frequency in the LTEE occur on an *uvrA* background (Ara−2 and Ara+6), whereas the third, in Ara−1, occurs on an *uvrC* background (fig. 3). The *mutT* allele in Ara−2 does not cause the characteristic *mutT* A:T→C:G hypermutator phenotype found in Ara−1 and Ara+6 (fig. 2), so its association with *uvrA* may be coincidental. However, the same *uvrA* substitution that goes to fixation with *mutT* in Ara+6 also occurs in a 40,000 generation isolate from the Ara−1 population called REL10939 (Tenaillon et al. 2016), which suggests that this particular *uvrA* allele may be beneficial in those contexts. Furthermore, it has been reported that *uvrA/mutT* and *uvrB*/*mutT* double knockouts have a substantially lower mutation rate than *mutT* knockouts, in the presence of hydrogen peroxide (Hori et al. 2007). Based on these observations, we hypothesize that the *mutT* alleles that successfully went to fixation in the LTEE may have evolved on an *uvrABC* genetic background that reduced the intensity of the *mutT* hypermutator phenotype.

### Gene-Orientation Mutation Bias Evolves in the LTEE

Several reports indicate that mutation rates differ between the leading and lagging strands of the DNA replication bubble (Lee et al. 2012; Paul et al. 2013). Potential causes include asymmetry in nucleotide composition around the replication origin (GC skew) (Marín and Xia 2008), context-dependent mutation rates that are asymmetric around the replication origin (Sung et al. 2015), and head-on collisions between the replication and transcription molecular machinery (Paul et al. 2013). Such reports motivated us to ask whether the LTEE metagenomics data showed evidence of gene-orientation mutation biases, such that genes oriented with (or against) the leading or lagging strand of DNA synthesis have different mutation rates.

**Fig. 5.**
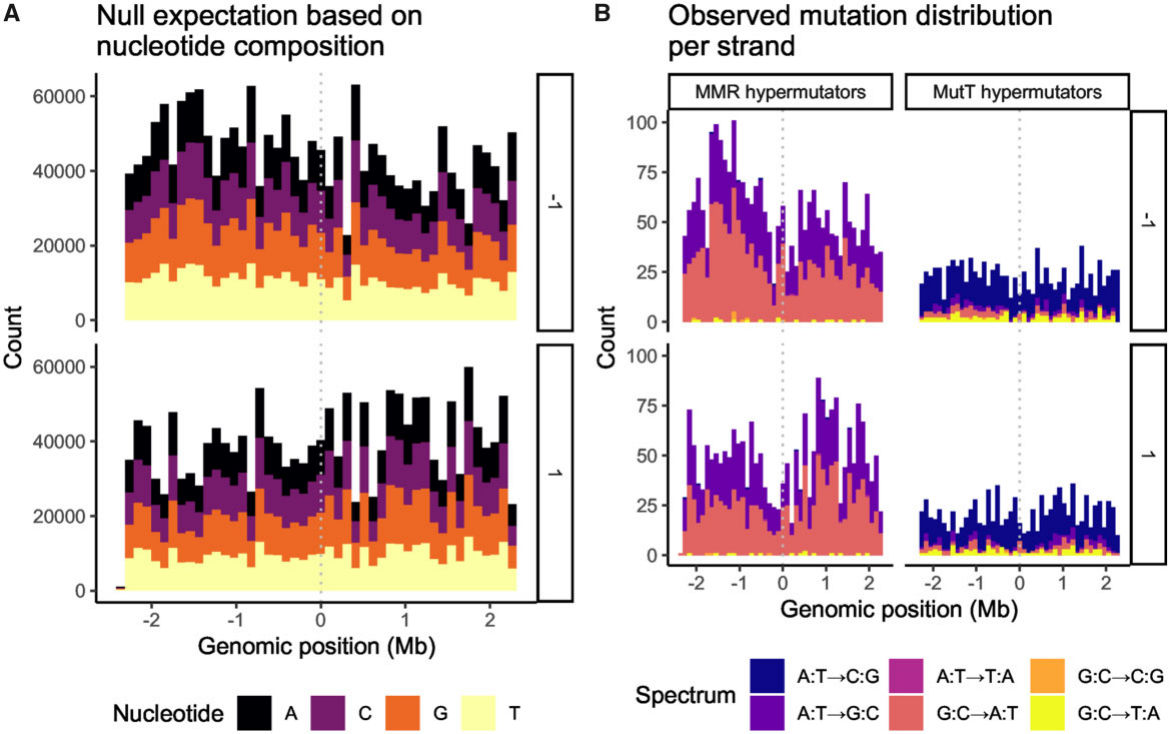
Gene-orientation mutation bias evolves in the LTEE. The *x* axis is the reference genome, centered on the replication origin, partitioned into 46 equally sized bins of ∼100 kb. In each labeled subfigure, top and bottom panels show genes occurring on each of the two strands of the chromosome, with the arbitrary labels 1 and −1. (*A*) The nucleotide composition of genes on the two strands of the chromosome of the LTEE ancestral clone REL606. (*B*) The genomic distribution of mutations within genes, summed over MMR-deficient LTEE populations (left panel) and MutT-deficient LTEE populations (right panel).

Our null expectation is that the distribution of synonymous mutations on each strand of the chromosome should be related to the amount of coding sequence on each strand (i.e., the density of genes multiplied by their length). Furthermore, the spectrum of nucleotide substitutions on each strand should reflect local G:C content in the ancestral LTEE clone REL606: for example, G:C→A:T substitutions should be more common in G:C-rich regions. Figure 5*A* shows this null expectation. Both the amount of coding sequence and G:C content per strand are asymmetric about the replication origin of REL606. At the replication origin, one DNA strand switches from leading to lagging, while its complement switches from lagging to leading. This switch occurs because DNA replication is bidirectional, such that two replisomes move in opposite directions from the replication origin. Even in the absence of gene-orientation mutation bias, figure 5*A* shows that some asymmetry in the distribution of synonymous mutations over the replication origin is expected.

The observed distributions of synonymous mutations on each strand of the chromosome are shown in figure 5*B*. We separately analyzed MMR- and MutT-deficient hypermutator populations. In both cases, the number of observed mutations significantly differs between genes oriented with or against the movement of the replisome, based on comparing the expected ratio of mutations to the observed ratio of mutations. The MMR-deficient hypermutator populations show significantly more gene-orientation mutation bias than expected (two-tailed binomial test: observed ratio of 2,066:2,664 mutations vs. expected ratio of 1,730,238:2,066,587 nucleotides; *P *=* *0.0090), whereas the MutT-deficient hypermutator populations show significantly less gene-orientation bias than expected (two-tailed binomial test: observed ratio of 947:1,033 mutations vs. expected ratio of 1,730,238:2,066,587 nucleotides; *P *=* *0.0446). Note that these calculations do not account for the characteristic mutation spectra of MMR- and MutT-deficient hypermutators (fig. 5*B*). For example, the extreme rate of A:T→C:G mutations seen in MutT-deficient hypermutators (Foster et al. 2015) should cause A:T rich genes to mutate faster than A:T poor genes.

### The Genomic Distribution of Observed Mutations in Ara+3 Shows a Strong, Symmetric Wave Pattern over the Origin of Replication

**Fig. 6.**
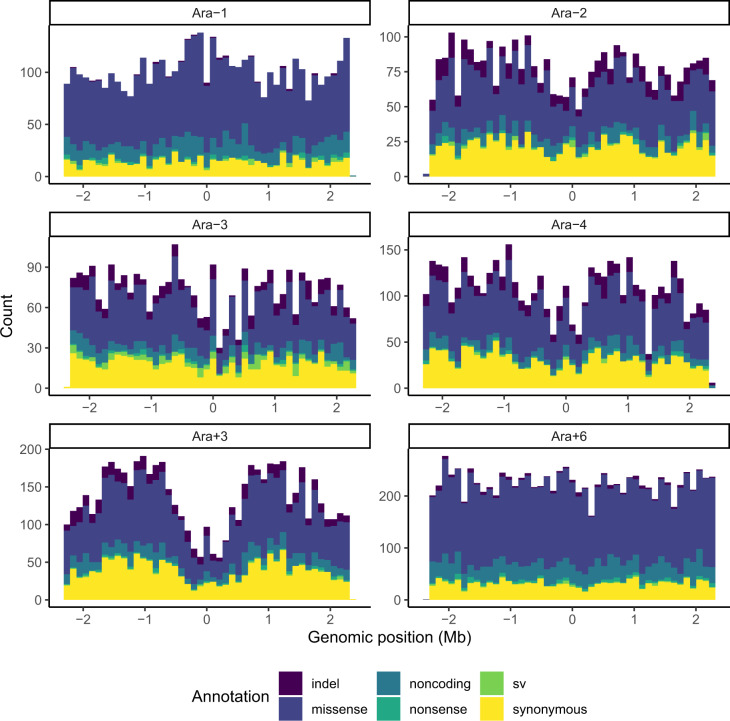
One hypermutator LTEE population, Ara+3, shows a strong wave pattern of mutation rate variation centered on the replication origin. Each panel shows the genomic distribution of mutations observed in each hypermutator LTEE population in the metagenomics data. The *x* axis is the reference genome, centered on the replication origin, partitioned into 46 equally sized bins of ∼100 kb. Indels are in purple, missense mutations are in dark blue, noncoding mutations are blue green, nonsense mutations are sea green, sv are green, and synonymous mutations are yellow.

**Fig. 7.**
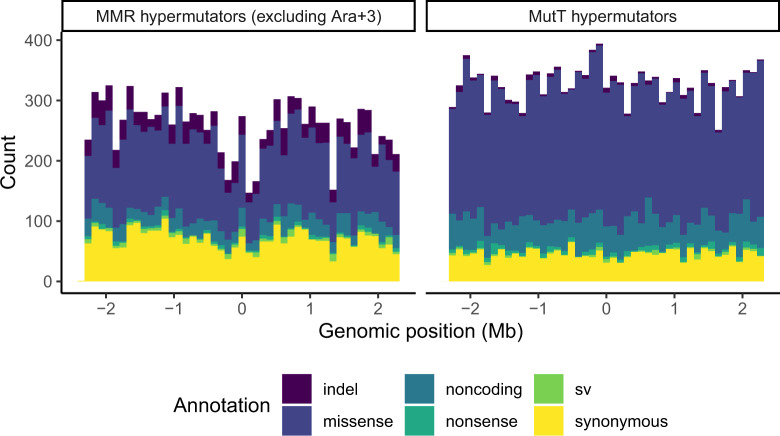
MMR-deficient LTEE populations (excluding Ara+3) show a weak wave pattern, whereas MutT-deficient LTEE populations show no wave pattern. The left panel shows the genomic distribution of mutations observed in Ara−2, Ara−3, and Ara−4. The right panel shows the genomic distribution of mutations observed in Ara−1 and Ara+6. The *x* axis is the reference genome, centered on the replication origin, partitioned into 46 equally sized bins of ∼100 kb. Indels are in purple, missense mutations are in dark blue, noncoding mutations are blue green, nonsense mutations are sea green, sv are green, and synonymous mutations are yellow.

Multiple studies (Sharp et al. 1989; Lang and Murray 2011; Foster et al. 2013; Dillon et al. 2018; Niccum et al. 2019) have reported correlations between local mutation rates and distance from the origin of replication. One hypermutator LTEE population, called Ara+3, shows a symmetric wave pattern reflected over *oriC* (fig. 6). Indeed, the genomic distribution of observed mutations in Ara+3 is significantly different from the genomic distribution of observed mutations summed over all hypermutator populations (two-sample Kolmogorov–Smirnov test: *D* = 0.0567, *P *<* *10^−14^). The wave in Ara+3 has a trough-to-peak ratio of ∼25:75 (fig. 6). Excluding Ara+3, the genomic distribution of observed mutations summed over the remaining MMR-deficient LTEE populations shows a weak wave pattern, whereas the populations with defects in *mutT* shows no evidence of the wave pattern (fig. 7). The genomic distribution of observed mutations in the MMR-deficient populations (excluding Ara+3) is significantly different from the genomic distribution of observed mutations in the MutT-deficient populations (two-sample Kolmogorov–Smirnov test: *D* = 0.040916, *P *<* *10^−9^).

### Evidence for Epistasis and Historical Contingency in the Evolution of DNA Topology

Why does a strong wave pattern only appear in Ara+3? Others have hypothesized that local chromatin structure affects local mutation rates (Foster et al. 2013; Niccum et al. 2019). Furthermore, DNA topology has evolved in parallel in the LTEE, and artificially increasing DNA supercoiling is beneficial under LTEE conditions (Crozat et al. 2005, 2010). Therefore, we hypothesized that mutations in genes that affect DNA topology might affect the wave pattern. To test this hypothesis, we examined the timing and distribution of mutations in *topA*, *fis*, and *dusB* (*yhdG*). We focused on these genes for several reasons. First, these loci show strong parallel evolution in the LTEE (Crozat et al. 2010). Second, introducing evolved alleles of *topA* and *fis* into the ancestral genome are sufficient to confer a fitness benefit as well as additive changes to DNA topology (Crozat et al. 2005). Finally, statistical analysis of the pattern of evolution for *dusB* and *fis* in the LTEE led to the discovery that *dusB* regulates *fis* expression (Crozat et al. 2005, 2010). We excluded synonymous mutations from this analysis. We counted both fixations and mutations destined for extinction, because many beneficial mutations go extinct in large asexual populations due to clonal interference (Gerrish and Lenski 1998; Lang et al. 2013; Levy et al. 2015; Maddamsetti, Lenski, et al. 2015; Ba et al. 2019).

**Fig. 8.**
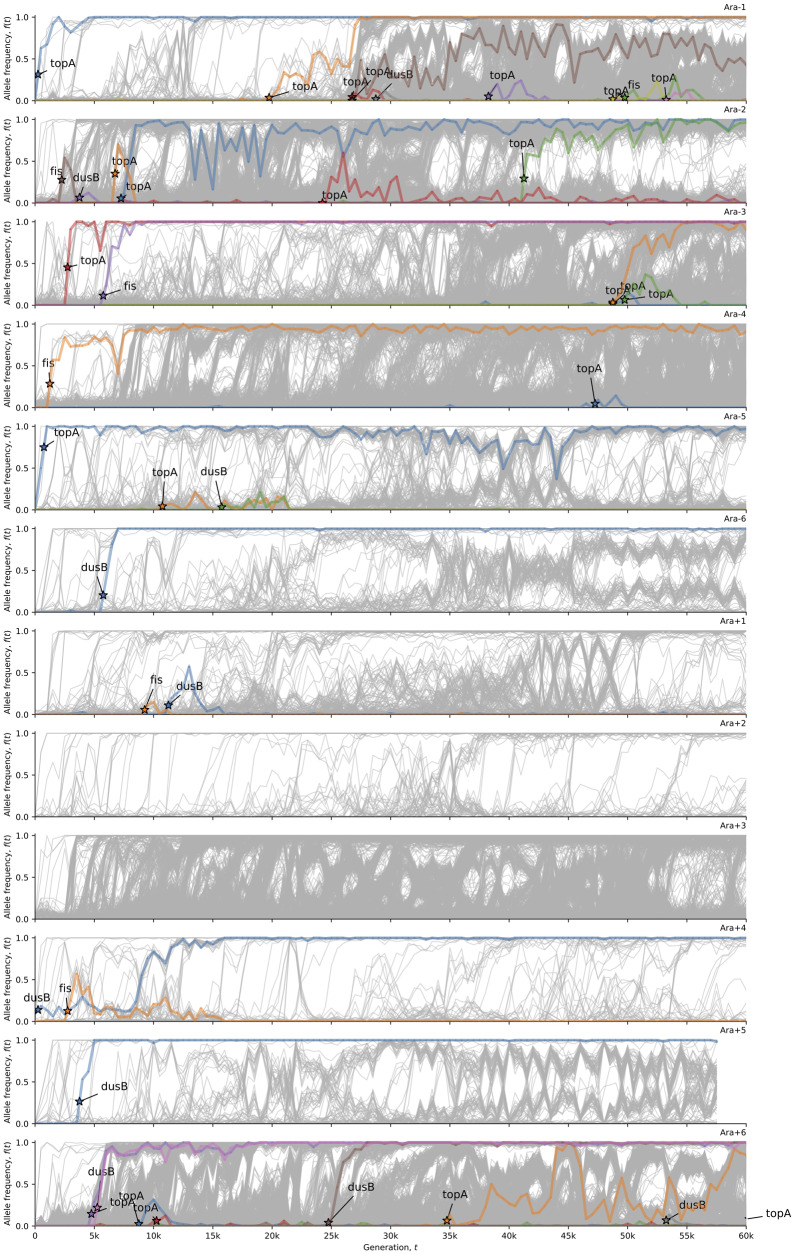
The strong wave pattern in Ara+3 anticorrelates with mutations (excluding synonymous changes) in the DNA topology genes *topA*, *fis*, and *dusB*. This visualization uses computer code written by Good et al. (2017). The allele frequency trajectories for all observed mutations in the 12 LTEE populations are shown in gray. The allele frequency trajectories of de novo mutations in *topA*, *fis*, and *dusB* (excepting synonymous mutations) are colored and labeled in each population.

All LTEE populations evolved missense, indel, or structural mutations in *topA*, *fis*, and *dusB* within the first 10,000 generations, except two: Ara+2 and Ara+3 (fig. 8). The timing and distribution of mutations in these genes across populations suggests epistasis and historical contingency (Good et al. 2017). The early arrival times for mutations in these genes suggests that there is an early, limited window of opportunity for those mutations to go to fixation. Quantitative evidence comes from Ara+3, which has no missense, nonsense, indel, or structural mutations in *topA*, *fis*, and *dusB* whatsoever, despite its strong hypermutator phenotype. The probability of this event is *P* = (1−(*t*/*g*))^*n*^, where *t* is the effective mutational target size, *g* is the length of the chromosome (*g = *4,629,812), and *n* is the number of observed missense, indel, and structural mutations in Ara+3 (*n *=* *4,368). Given the wave pattern in Ara+3, the effective mutational target size of *topA*, *fis*, and *dusB* could be smaller than their combined physical target size (3,861 bp), say if they occurred in the trough of the wave. To take this into account, we partitioned the chromosome into bins, counted mutations per bin, and calculated the effective mutational target size by multiplying the physical target size (length) of *topA*, *fis*, and *dusB* by the number of mutations per base pair in their respective bins. These genes are significantly depleted of mutations in Ara+3, for bin sizes ranging from 100 kb to the entire chromosome (one-tailed randomization tests with 10,000 bootstraps: *P *<* *0.05 in all cases).

The distribution of synonymous mutations in *topA*, *fis*, and *dusB* across the LTEE populations is interesting (supplementary fig. S4 and Supplementary Material online). A single, synonymous A312A substitution in *dusB* went to fixation at ∼4,000 generations in Ara+3, simultaneously with alleles in the MMR genes *mutS* and *mutH* that apparently caused the early hypermutator phenotype in this population. No other synonymous mutations in *dusB* are observed in Ara+3. Furthermore, there is evidence of parallel evolution at this particular position in *dusB*. The same synonymous mutation occurs in Ara+6, and another synonymous mutation, one base pair downstream in the next codon, is the only synonymous mutation in *topA*, *fis*, or *dusB* observed in Ara−2 (supplementary fig. S4, Supplementary Material online). This parallelism suggests that positive selection may be acting on these synonymous variants. Overall, it is striking how few synonymous mutations in *topA*, *fis*, and *dusB* occur in the hypermutator LTEE populations, which implies that synonymous variants in these genes may not be evolving neutrally. Indeed, STIMS (Maddamsetti and Grant 2020) finds a significant signal of purifying selection on synonymous mutations in *topA*, *fis*, and *dusB* in Ara−1 and Ara−3 (one-tailed randomization test with 10,000 bootstraps: *P *<* *0.0001).

We also examined the genes that encode the nucleoid-binding protein HU and the terminus-organizing protein MatP, as deletions of these loci were shown to affect the wave pattern (Niccum et al. 2019). Notwithstanding the relevance of HU and MatP in Niccum et al. (2019), these genes show limited evidence of parallel evolution in the LTEE (supplementary fig. S5, Supplementary Material online).

### Synonymous Nucleotide Diversity in Natural *E. coli* Populations Does Not Predict Mutation Rate Variation in the LTEE

Finally, we used the LTEE metagenomic data to revisit previous work, which found that the distribution of synonymous mutations in the LTEE does not reflect patterns of synonymous variation among natural *E. coli* isolates (Maddamsetti et al. 2015). During our reanalysis, we found a potential coding error affecting the results of the Kolmogorov–Smirnov test reported in that paper. Therefore, we used Poisson regression to ask whether the estimates of synonymous nucleotide diversity *θ*_s_ published in Martincorena et al. (2012), when treated as gene-specific estimates of the point-mutation rate per base pair, predict the distribution of synonymous mutations observed in the LTEE. A null model in which mutations occur uniformly over the chromosome (Akaike’s Information Criterion, AIC = 8,529.6) fits the data far better than the *θ*_s_ model (AIC = 9,171.3). When we fit both models to Ara+3, we again find that the null model is better than the *θ*_s_ model at predicting the observed distribution of synonymous mutations (AIC = 2,168.2 for null model vs. AIC = 2,190.8 for *θ*_s_ model). This finding validates the conclusions reported in Maddamsetti et al. (2015), despite the potential problems in that analysis.

## Discussion

By examining the distribution of observed mutations over more than 60,000 generations of the LTEE (Good et al. 2017), we find that mutation rates and biases have diverged idiosyncratically, despite identical abiotic conditions. One LTEE population, Ara+3, shows strong evidence of the wave pattern in mutation rate variation. Similar patterns have been seen in mutation accumulation experiments with MMR-deficient strains of *E. coli* as well as in *Vibrio* bacteria (Dillon et al. 2018; Niccum et al. 2019). Our result shows that genomic biases in mutation rates evolve dynamically on laboratory timescales. It is likely that the identity and effects of many hypermutator and antimutator alleles in the LTEE remains unknown. For instance, we do not know what alleles, if any, cause the apparent late decrease in mutation rate seen in Ara+3. Experiments are needed, both to discover those unknown alleles, and to test for genetic interactions that modulate mutation rates in the LTEE, as we have hypothesized for alleles of *uvrABC* and *mutT*.

The divergence in the rates, biases, and spectra of mutations across replicate populations in this simple long-term evolution experiment makes one wonder about the scope of natural variation in mutation rates, biases, and spectra. An evolution experiment with replicate mouse microbiomes has indicated that microbial evolution in the gut is probably characterized by long-term maintenance of intraspecies genetic diversity, including mutation rate polymorphism (Ramiro et al. 2020). Phylogenomic studies have also found extensive evidence for horizontal gene transfer in DNA repair genes (Denamur et al. 2000), which suggests that polymorphism in DNA repair genes may cause extensive natural variation in mutation and recombination rates within and across bacterial (meta-) populations and communities.

We find statistical evidence for historical contingency and epistasis in the evolution of DNA topology in the LTEE, and for Ara+3 in particular. These findings suggest a relationship between local DNA topology and local mutation rate variation, consistent with the experiments reported by Niccum et al. (2019). These findings immediately suggest the need for experiments to test whether local DNA topology causes local mutation rate variation, and to test whether local DNA topology affects strand-specific and gene-orientation mutation biases.

A comparison of synonymous genetic variation estimated from natural *E. coli* isolates to the distribution of observed synonymous mutations in the LTEE confirms the conclusion of earlier work (Maddamsetti et al. 2015) using richer data, and is consistent with other reports as well (Lee et al. 2012; Chen and Zhang 2013; Lynch et al. 2016). In sum, gene-specific variation in synonymous nucleotide diversity *θ*_s_, estimated from natural isolates of *E. coli*, does not predict the genomic distribution of synonymous mutations observed in the LTEE. In any case, the other results that we have presented, in addition to prior reports (Foster et al. 2013; Paul et al. 2013; Sung et al. 2015; Jee et al. 2016; Niccum et al. 2019), strongly indicate that mutation rates vary over the *E. coli* chromosome.

These results add to the robust debate on the causes and consequences of mutation rate evolution. It is clear that a deeper understanding of the relationships among chromatin structure, genomic variation in mutation and recombination rates, and natural selection, and their consequences for short- and long-term genome evolution, will be a fruitful goal for further research.

## Materials and Methods

Preprocessed LTEE metagenomic data, and associated analysis and visualization code was downloaded from: https://github.com/benjaminhgood/LTEE-metagenomic. Analysis codes are available from: https://github.com/rohanmaddamsetti/LTEE-purifying-selection/blob/master/mutation-rate-analysis.R and https://github.com/rohanmaddamsetti/LTEE-purifying-selection/blob/master/metagenomics-library.R. We systematically examined DNA repair genes in *E. coli* (Eisen and Hanawalt 1999; Lee et al. 2016; Deatherage et al. 2018), as well as annotated DNA polymerases, and other proteins of the replisome. A table of these genes and their annotations is in supplementary data file 1, Supplementary Material online. We cross-checked the evolutionary dynamics of alleles of these genes in the LTEE metagenomic data against the observed changes in mutation rates and spectra in each LTEE population. We also examined the LTEE genomic data (Tenaillon et al. 2016) for mutations in these genes, using the R Shiny web app interface at www.barricklab.org/shiny/LTEE-Ecoli. In this manner, we curated a list of putative hypermutator and antimutator alleles in the LTEE (table 1). Those alleles, and alleles of other genes in their respective DNA repair pathways, are shown in figures 3 and 4. Figure 3 shows the evolutionary dynamics of alleles in genes encoding base excision repair, nucleotide excision repair, and degradation of oxidized nucleotide triphosphates. Figure 4 shows the evolutionary dynamics of alleles in genes encoding DNA MMR. Data sets and analysis codes to replicate the findings and figures in this paper are available on the Dryad Digital Repository (DOI: https://doi.org/10.5061/dryad.kprr4xh2z.).

## Supplementary Material

evaa178_Supplementary_Data
