# Supplementary material for: Divergent Evolution of Mutation Rates and Biases in the Long-Term Evolution Experiment with Escherichia coli
Source: Genome Biol Evol. 2020 Aug 27;12(9):1591–603. doi: 10.1093/gbe/evaa178 (PMC7523724; doi:10.1093/gbe/evaa178)
Supplement: evaa178_Supplementary_Data [file evaa178_supplementary_data.zip › LTEE-mutation-bias-v3-Supplementary-Information.docx]

**SUPPLEMENTARY INFORMATION:**

**Supplementary Figure S1. Allele frequency trajectories for all observed point mutations in the twelve LTEE populations.** This visualization uses computer code written by Good et al. (2017). The allele frequency trajectories for all observed mutations in the twelve LTEE populations are shown in grey. Stars indicate the time (and allele frequency) at which mutations are reliably estimated to appear in the time series.

**
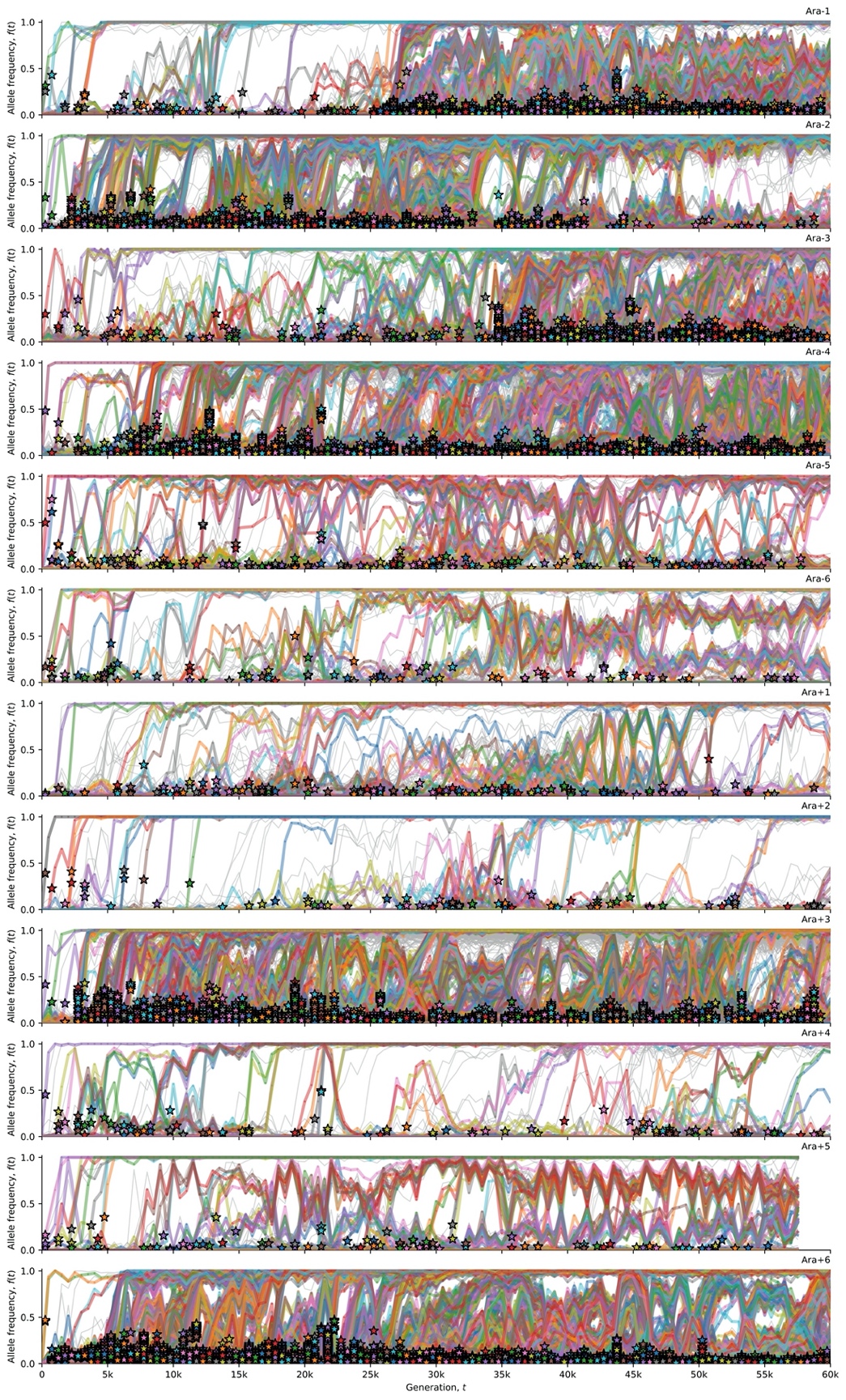
**

**Supplementary Figure S2. Allele frequency trajectories for all observed indel mutations in the twelve LTEE populations.** This visualization uses computer code written by Good et al. (2017). The allele frequency trajectories for all observed mutations in the twelve LTEE populations are shown in grey. Stars indicate the time (and allele frequency) at which mutations are reliably estimated to appear in the time series.

**
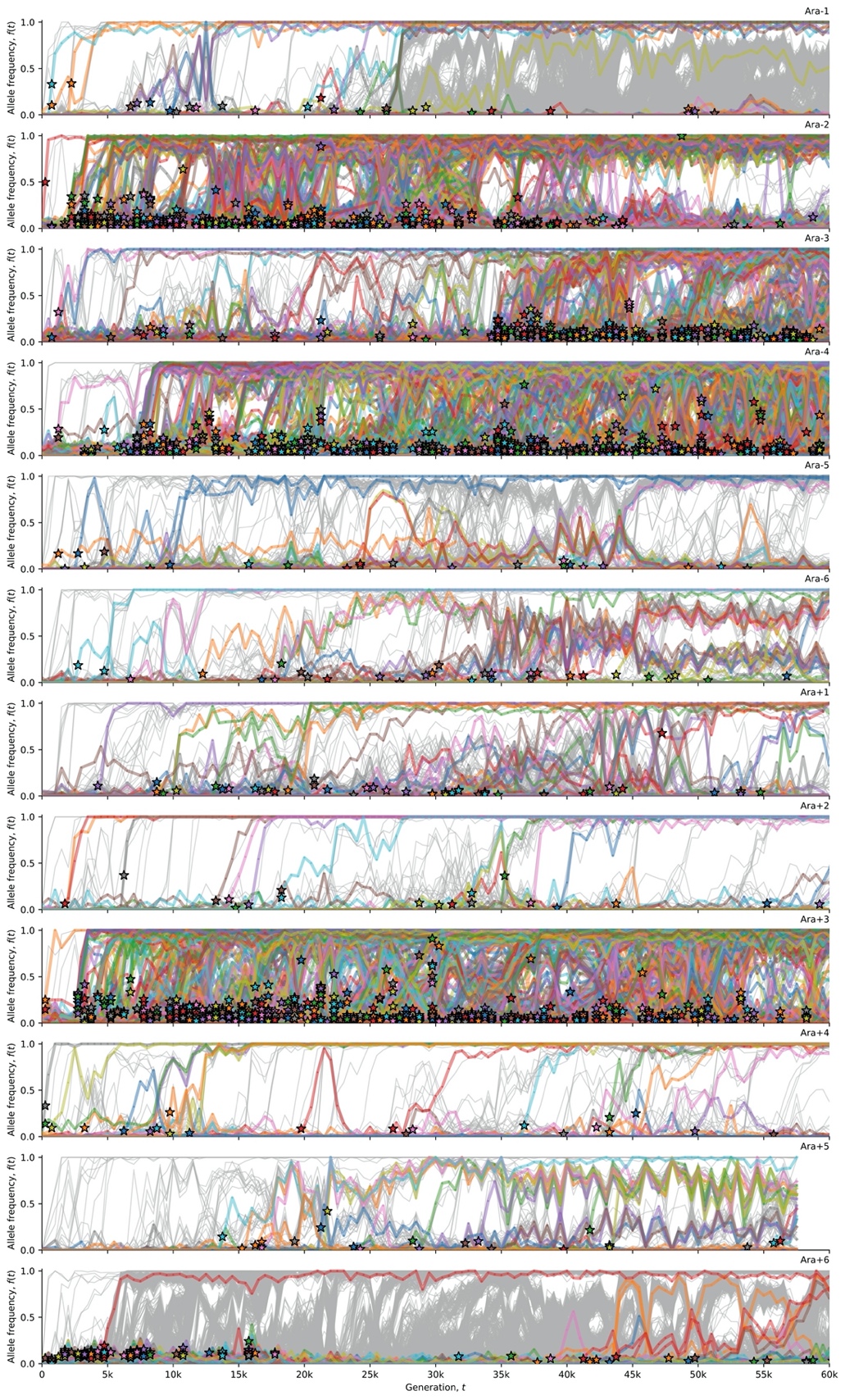
**

**Supplementary Figure S3. Allele frequency trajectories for all observed structural mutations in the twelve LTEE populations.** This visualization uses computer code written by Good et al. (2017). The allele frequency trajectories for all observed mutations in the twelve LTEE populations are shown in grey. Stars indicate the time (and allele frequency) at which mutations are reliably estimated to appear in the time series.

**
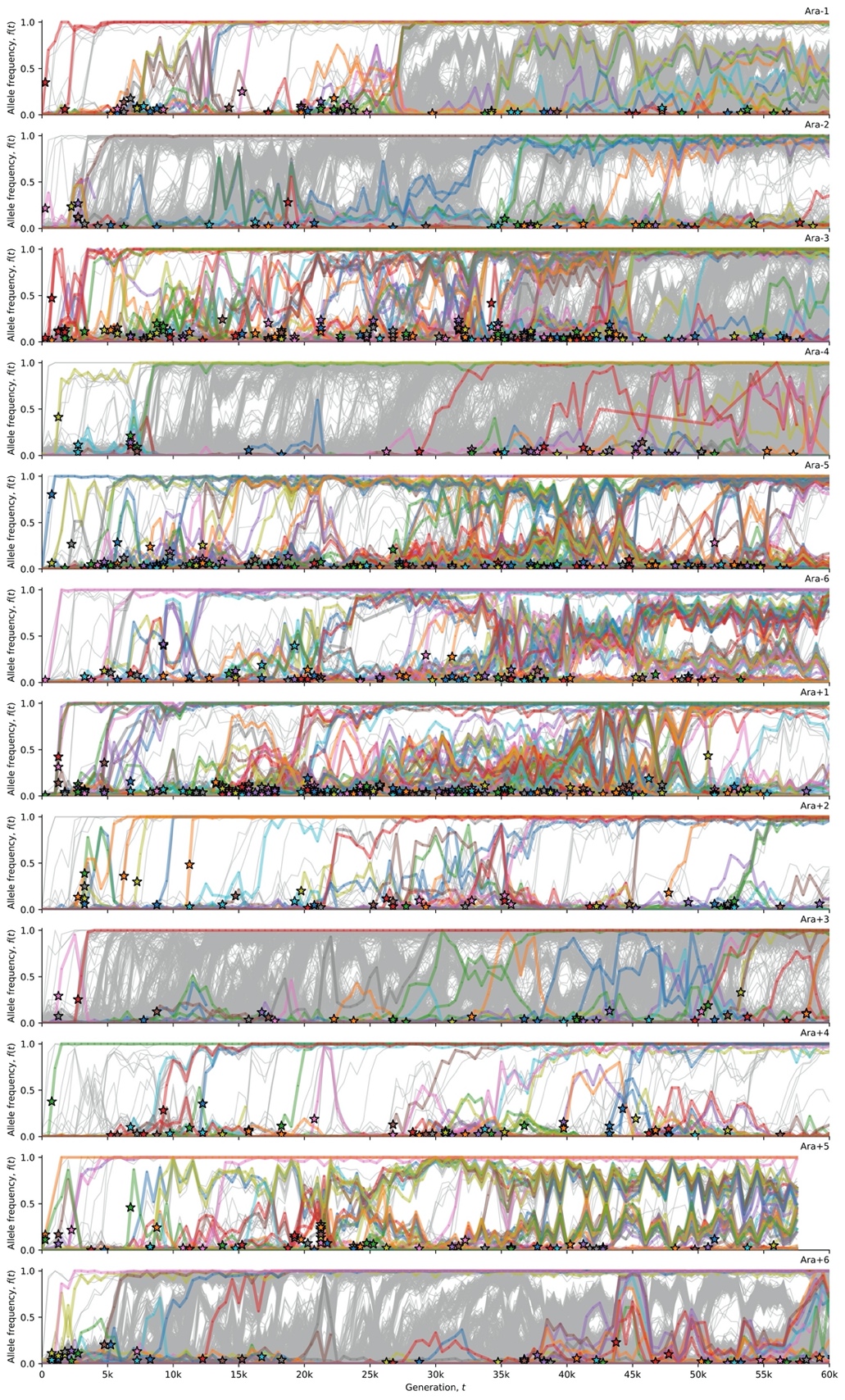
**

**Supplementary Figure S4. Synonymous mutations in the DNA topology genes *topA, fis,* and *dusB*.** This visualization uses computer code written by Good et al. (2017). The allele frequency trajectories for all observed mutations in the twelve LTEE populations are shown in grey. The allele frequency trajectories of *de novo* synonymous mutations in *topA, fis,* and *dusB* are colored and labeled in each population.

**
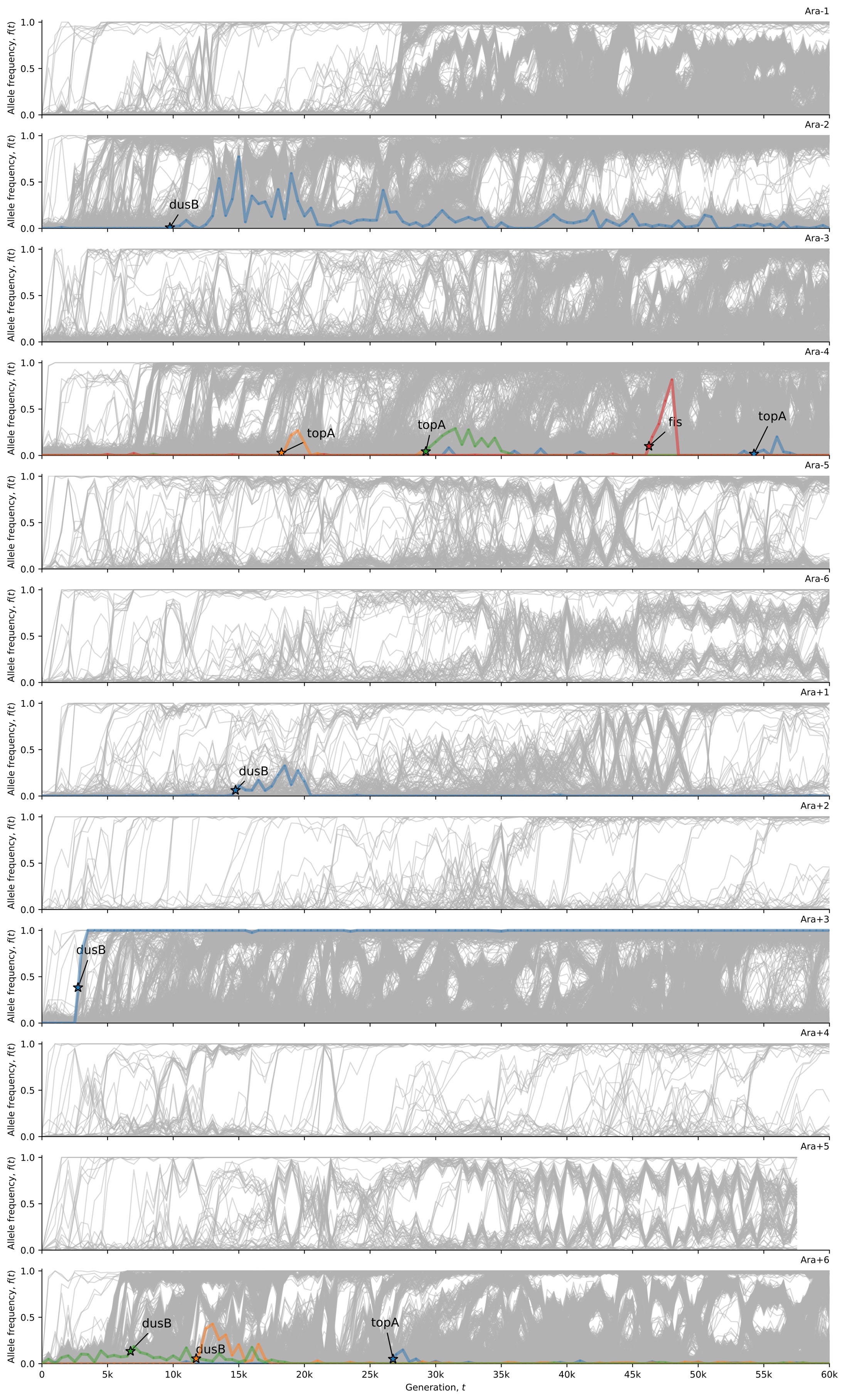
**

**Supplementary Figure S5. Mutations in the DNA topology genes *hupA*, *hupB,* and *matP* (excluding synonymous mutations).** This visualization uses computer code written by Good et al. (2017). The allele frequency trajectories for all observed mutations in the twelve LTEE populations are shown in grey. The allele frequency trajectories of *de novo* mutations in *hupA, hupB,* and *matP* are colored and labeled in each population.

**
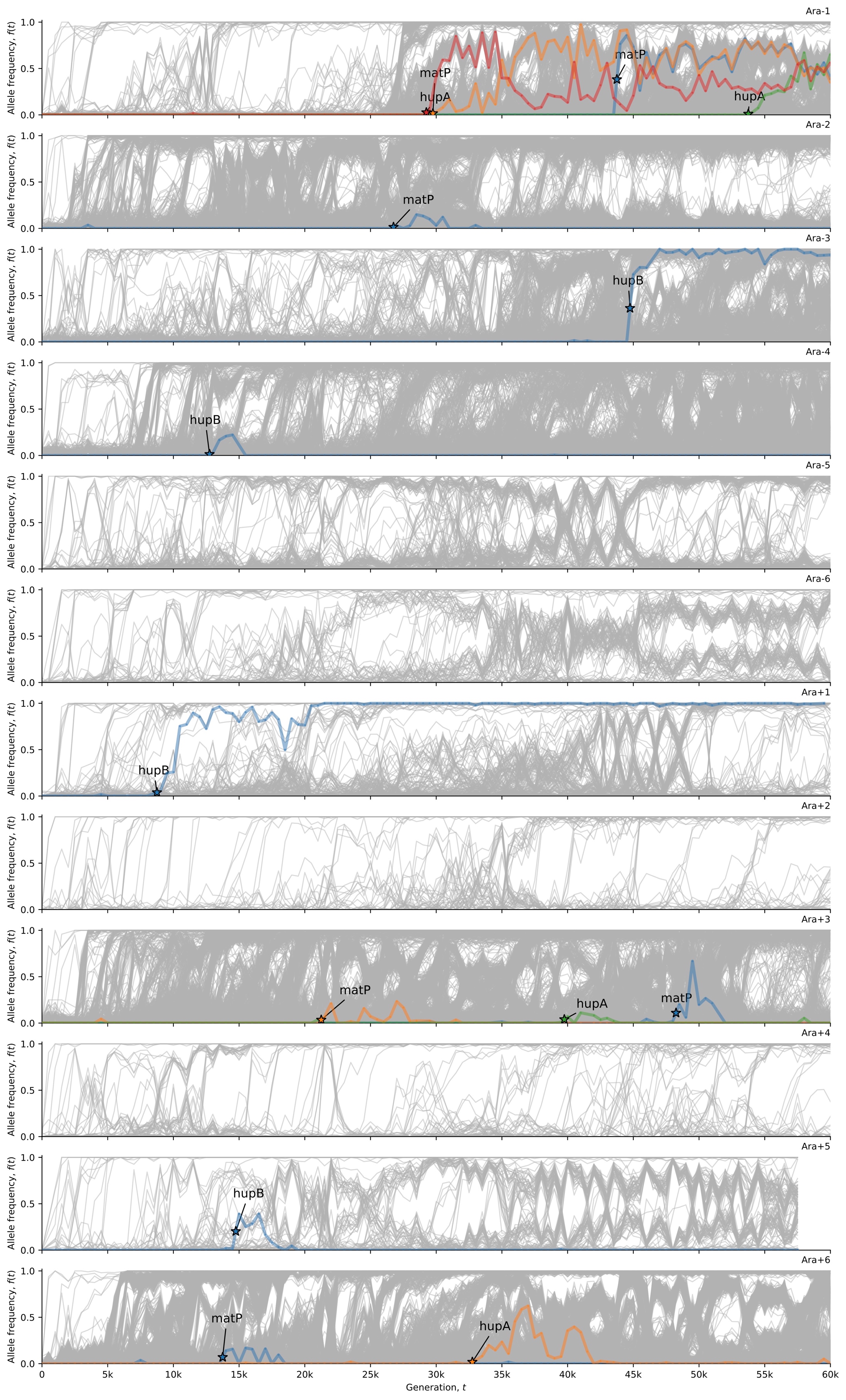
**

**Supplementary File 1: Annotated DNA repair and replication genes in *Escherichia coli*.**
